# Multiple Fano resonances with flexible tunablity based on symmetry-breaking resonators

**DOI:** 10.3762/bjnano.10.236

**Published:** 2019-12-11

**Authors:** Xiao bin Ren, Kun Ren, Ying Zhang, Cheng guo Ming, Qun Han

**Affiliations:** 1 School of Science, Tianjin University of Science and Technology, Tianjin 300222, China; 2 College of Precision Instrument and Opto-electronics Engineering; Key Laboratory of Opto-electronics Information Technology, Ministry of Education, Tianjin 300072, China

**Keywords:** multiple Fano resonance, off-centered ring resonators, plasmonic waveguide, surface plasmon polaritons, symmetry-breaking, tunable resonance

## Abstract

A symmetry-breaking nanostructure is proposed to achieve multiple Fano resonances. The nanostructure consists of an asymmetric ring resonator coupled to a plasmonic waveguide. The broken symmetry is introduced by deviating the centers of regular ring. New resonant modes that are not accessible through a regular symmetric ring cavity are excited. Thus, one asymmetric cavity can provide more than one resonant mode with the same mode order. As a result, the interval of Fano resonances is greatly reduced. By combining different rings with different degrees of asymmetry, multiple Fano resonances are generated. Those Fano resonances have different dependences on structural parameters due to their different physical origin. The resonance frequency and resonance peak number can be arbitrarily adjusted by changing the degree of asymmetry. This research may provide new opportunities to design on-chip optical devices with great tuning performance.

## Introduction

Fano resonances originate from the interference of a discrete state and a continuum state [1–2]. Contrary to symmetric Lorentz line shapes, a Fano profile is sharp and asymmetric. Due to this unique line shape and the large induced field enhancements, Fano resonances can potentially applied in sensors [3–4], demultiplexers [5], lasers [6], filters [7], and photoswitches [8].

Various structures have been designed to realize Fano resonances, including metallic nanoclusters [9–10], individual plasmonic dolmen nanocavity [11], ring/disk cavities [12–13], and metamaterials and metasurfaces [14–16]. In particular, as an important geometry, waveguide–cavity stuctures are investigated at different platforms aiming for Fano resonance. Fano-type transmission phenomenona were observed in photonic crystal (PhC) waveguide–cavity systems [17–18]. The PhC waveguide is a line defect formed by removing a row of rods or air holes. The cavity is a point defect formed by reducing the radius of a single rod or removing an air hole. Fano profiles appear when partially reflecting elements are placed in the PhC waveguide. The physical origin of the Fano profile is the coupling of a discrete state (provided by cavity) to a continuum (provided by the waveguide with partially reflectance).

In addition to PhC waveguides, metal–dielectric–metal (MDM) waveguides are very attractive for researchers because they can support surface plasmon polaritons (SPPs) and allow for the control of light at the subwavelength scale. MDM waveguides provide an effective approach to chip-scale photonic components [19–22]. Fano resonances have been obtained in MDM-based waveguide–cavity coupled systems [23–24]. In recent years, multiple Fano resonances have aroused interest [25–27]. Compared with a single Fano resonance, multiple Fano resonances have more versatile and flexible applications, such as self-reference and multichannel sensing [25–26].

The generation of multiple Fano resonances is attributed to the existence of multiple discrete states. In most of the published papers, high-order modes are excited in the same cavity [23,28]. Discrete states are provided by different modes from the same cavity, thus the resulting multiple Fano resonances are connected to the different orders of the mode. As a result, the frequency interval of the Fano resonances is usually very large, ranging over hundreds of nanometers. Recently, multiple Fano resonances were achieved with the aid of different cavities that provided several discrete states [26,29–30]. For instance, two U-shaped resonators were designed in order to obtain independent dual resonances [30].

In this paper, multiple Fano resonances are investigated and demonstrated in MDM waveguide–cavity coupled systems. A symmetry-breaking ring cavity is proposed. Discrete states are obtained in the same cavity without exciting high-order cavity modes. Because of the equal mode order, the interval of the resonant frequency is reduced. The manipulation of Fano resonances is further discussed by adjusting the degree of asymmetry. More Fano peaks appear in the transmission spectrum after combining two symmetry-breaking cavities with different dimensions.

## Structure and Theory

**Figure 1:**
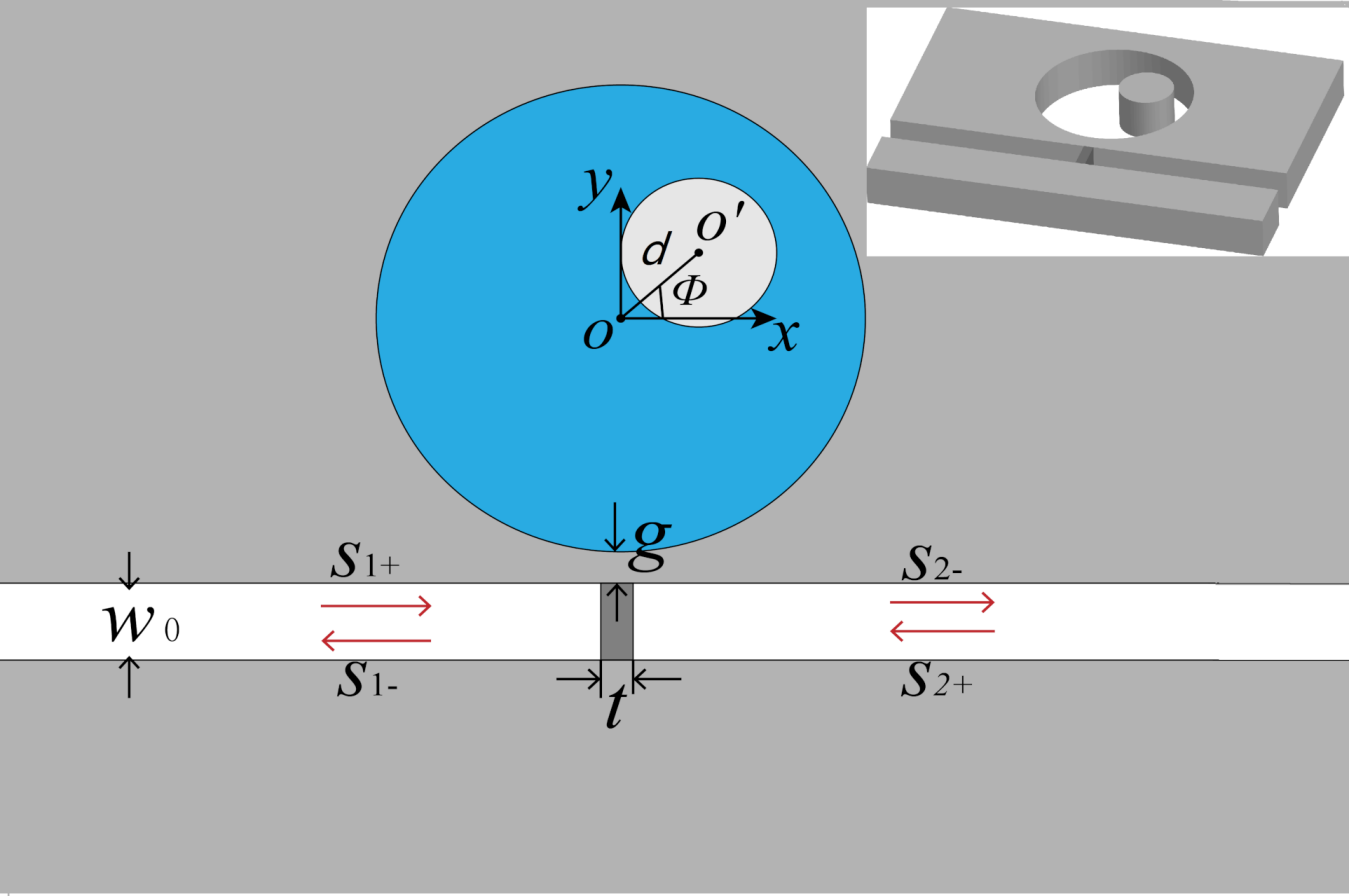
Schematic diagram of an off-centered ring resonator (OCRR) side-coupled to the MDM waveguide. The inset is the 3D view. The outer and inner radius of the OCRR are *R* and *r*, respectively. The deviation of the center points is denoted by *d* (i.e., *OO*′). The deviation angle is described by ϕ, which is the angle between the center connection and the *x*-axis. The width of waveguide is *W*_0_. The thickness of the metal wall in the waveguide is *t*. The coupling distance between the disk resonator and the upper stub is *g*.

Figure 1 shows the schematic diagram of the MDM waveguide–cavity coupled system. The inset is the 3D view. The asymmetric cavity that we designed is an off-centered ring resonator (OCRR). *O* and *O*′ are the center of the outer and the inner circle, respectively. The distance between *O* and *O*′ is denoted by *d*. When *d* = 0, the cavity is a regular ring. When *d* ≠ 0, an off-centered ring with symmetry breaking is obtained. The deviation angle between the center connection and the *x*-axis is described by ϕ. The distance *d* and the angle ϕ are parameters that are closely related to the degree of asymmetry. The radius of the outer and the inner ring cavity are *R* and *r*, respectively. The OCRR is side-coupled to the waveguide with the gap *g*. The width of the MDM waveguide is *W*_0_. A metal wall with the thickness *t* is placed inside the MDM waveguide.

The grey parts in Figure 1 stand for metal (ε_m_). Both the metal wall and the background metal are silver the complex relative permittivity of which is characterized by the Drude model,


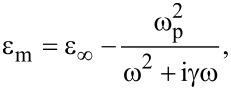


where ω is the angular frequency of the incident light. The other parameters are ε_∞_ = 3.7, bulk plasma frequency ω_p_ = 1.38 × 10^16^ Hz, damping frequency γ = 2.73 × 10^13^ Hz. The dielectric in the waveguide is air. The ring resonator is filled with a dielectric with the constant ε_d_.

Temporal coupled-mode theory (CMT) is used to analyze the transmission characteristics [25,31]. For the studied waveguide–resonator coupled system, the coupling coefficients between ring resonator and input (output) waveguide are denoted by κ_1_ (κ_2_). θ_1_ (θ_2_) are the phase shift of the coupling coefficient between ring resonator and input (output) waveguide. The decay rate due to the internal loss in the resonators is κ*_i_*. The amplitudes of the incoming and outgoing waves in the waveguide are denoted by *S_i_*_+_ and *S_i_*_−_ (*i* = 1, 2).

The time evolution of the normalized amplitude *a* of ring resonator can be expressed as

[1]
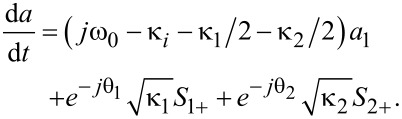


According to energy conservation, the amplitudes of the incoming and the outgoing waves in coupled waveguides satisfy the following relationships:

[2]
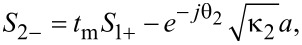


where *t*_m_ is the transmission coefficient for SPPs passing through the waveguide with a metal wall inside. It represents the direct coupling between input waveguide and output waveguide. If there is no metal wall in the waveguide *t*_m_ is almost equal to 1. Equation 2 can then be rewritten as 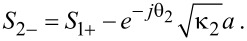 If the transmission is completely forbidden due to metal wall, then *t*_m_ = 0.

When an optical wave with frequency ω is launched only from the left port of the waveguide (*S*_2+_ = 0), the transmission of waveguide–resonator coupled system can be derived as:

[3]
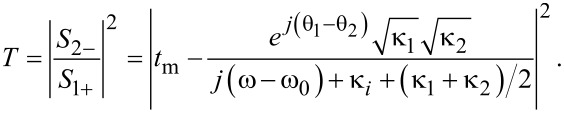


If the width of the input waveguide is the same as the width of the output waveguide, then θ_1_ = θ_2_ and κ_1_ = κ_2_. When there is no metal wall (corresponding to *t*_m_ = 1), the transmission efficiency through the waveguide–resonator system is described by:

[4]
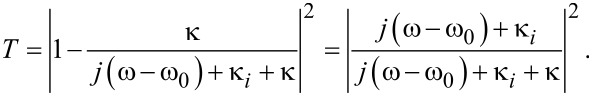


The above expression agrees with Equation 3 in [32], which deals with a waveguide with a side-coupled nanocavity. Equation 4 shows that at the resonance frequency ω_0_ the transmission reaches minimum: *T*_min_ = |κ*_i_*/(κ*_i_* + κ)|^2^. This means there would be a narrow transmission dip. If there is no internal loss κ*_i_*, we have *T*_min_ = 0. When the transmission coefficient *t*_m_ is relatively low and closes to zero, Equation 3 can be expressed as:

[5]
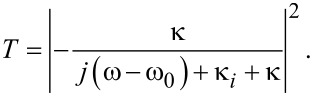


Equation 5 shows that the maximum transmission *T*_max_ happens when ω = ω_0_: *T*_max_ = |κ/(κ*_i_* + κ)|^2^. The transmission spectrum has a peak around the resonance frequency ω_0_. If there is no internal loss κ*_i_*, we have *T*_max_ = 1.

## Results and Discussion

Numerical simulations were performed by using COMSOL Multiphysics. The width of the waveguide is *W*_0_ = 50 nm, the gap between waveguide and resonator is *g* = 10 nm. The outer and inner radius of ring are *R* = 155 nm and *r* = 55 nm, respectively. The deviation angle is ϕ = 0° and the deviation distance is *d* = 0.

**Figure 2:**
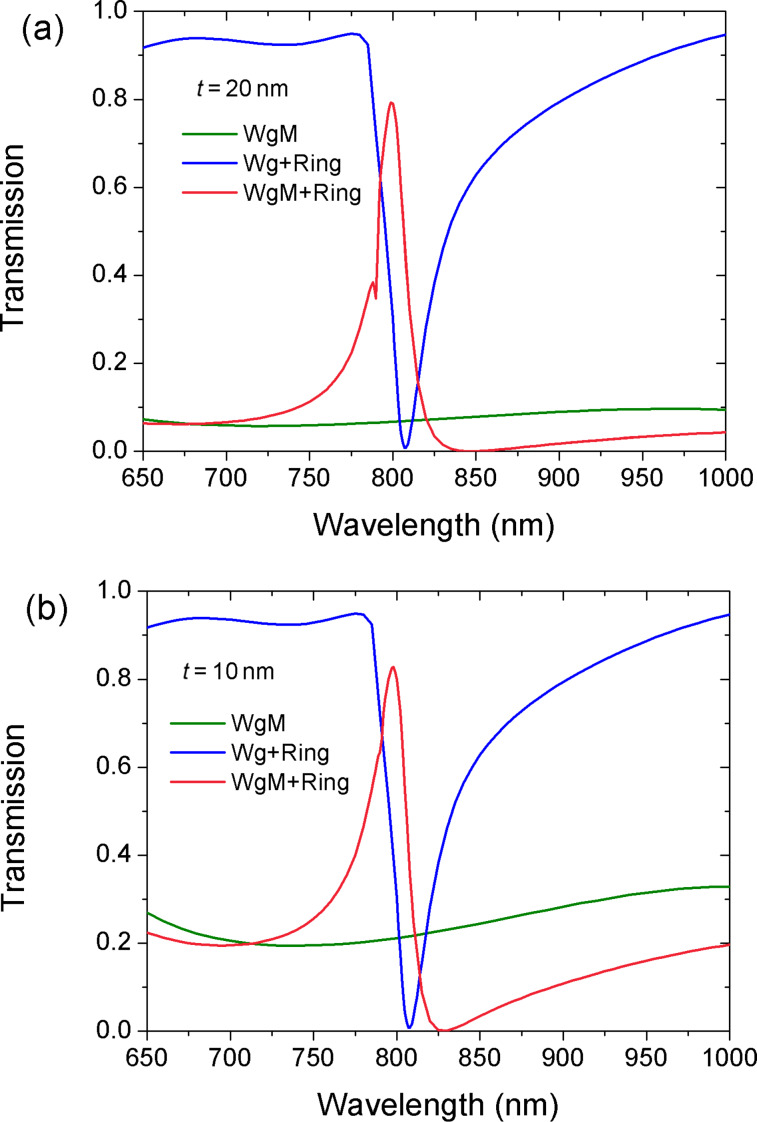
Transmission spectra for different structures. (a) *t* = 20 nm, and (b) *t* = 10 nm. The other parameters are *R* = 155 nm, *r* = 55 nm and ϕ = 0°.

Figure 2 shows the transmission spectra of the designed waveguide–resonator coupled system. Different values of the thickness of the metal wall, *t*, were studied. When a metal wall is placed in a MDM waveguide, the transmittance is low over a wide spectral range (green curve). The continuum state is provided by the waveguide with a metal wall inside (WgM). When a ring is side-coupled to an ordinary waveguide (Wg+Ring), a narrow dip at 807 nm appears in the transmission spectrum (blue curve). This dip means that the discrete state can be provided by the side-coupled ring cavity. For the system consisting of a ring and a waveguide with inserted metal wall (WgM+Ring), a transmission peak appears at around 800 nm (red curve) in an asymmetric transmission profile. When metal thickness is *t* = 10 nm, the asymmetric line shape is more obvious, as shown in Figure 2b. We consider this resonance a Fano resonance that arises from the interference of a resonant ring mode and a waveguide mode. From Figure 2, we infer that the position of the Fano peak is determined more strongly by the ring cavity.

**Figure 3:**
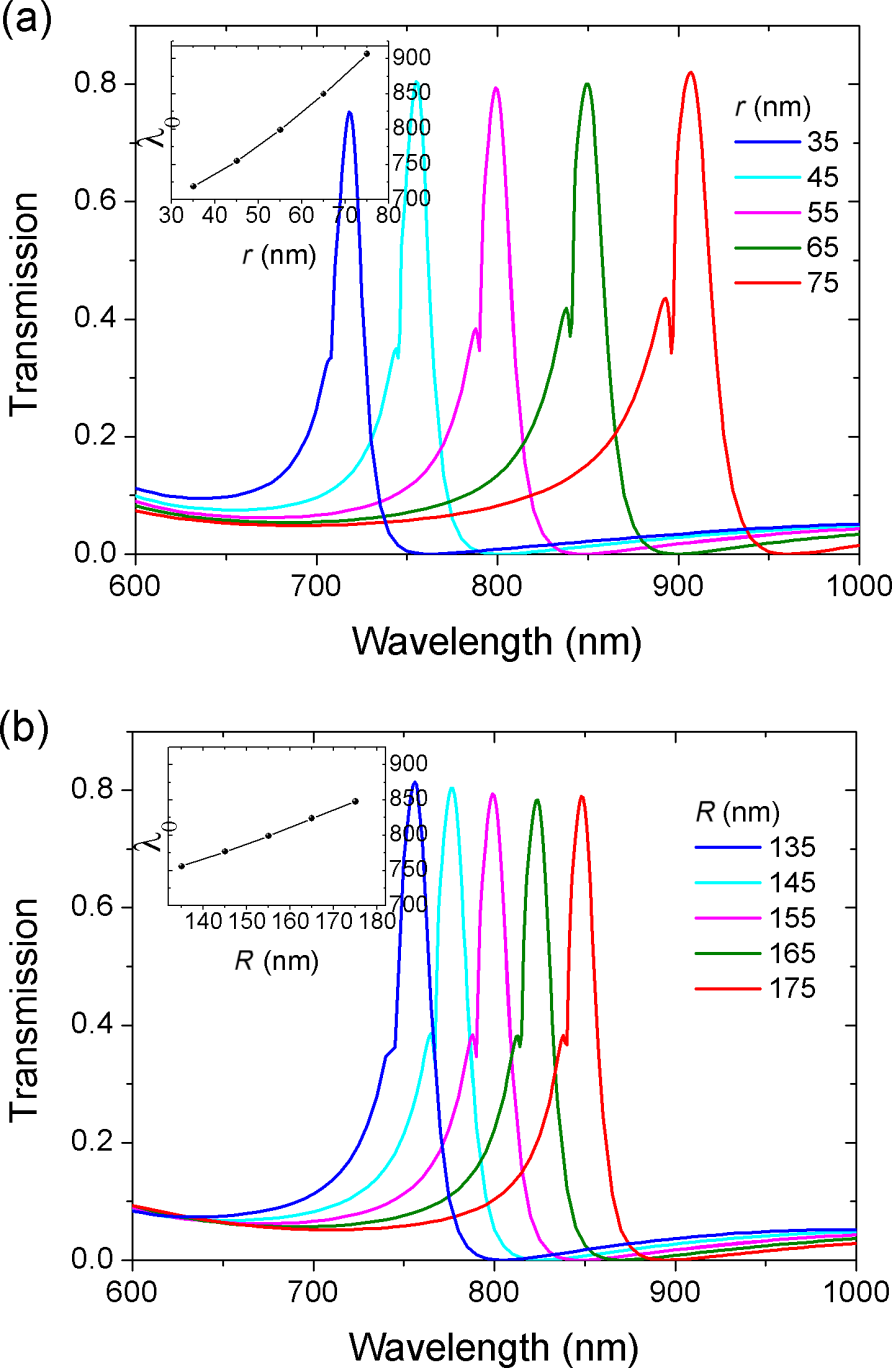
Transmission spectra at different structural parameters: (a) inner radius *r*, (b) outer radius *R*. The inset shows the resonance wavelength λ_0_ as a function of radius *r*.

In the following, the influence of the ring cavity on the Fano resonance is investigated. The evolution of the transmission spectra as a function of the inner radius is given in Figure 3a. The transmission peaks move to longer wavelengths with the increase of *r*. The inset shows the resonance wavelength λ_0_ as a function of *r*. The resonance wavelength is proportional to *r*. This can be explained by the resonance function. The resonance wavelengths are determined by:

[6]
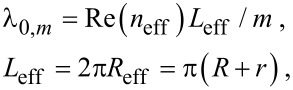


where *L*_eff_ is the effective length of ring resonator. *m* is an integer corresponding to the mode order, and *n*_eff_ is the effective index, which can be obtained from dispersion equation [33].

**Figure 4:**
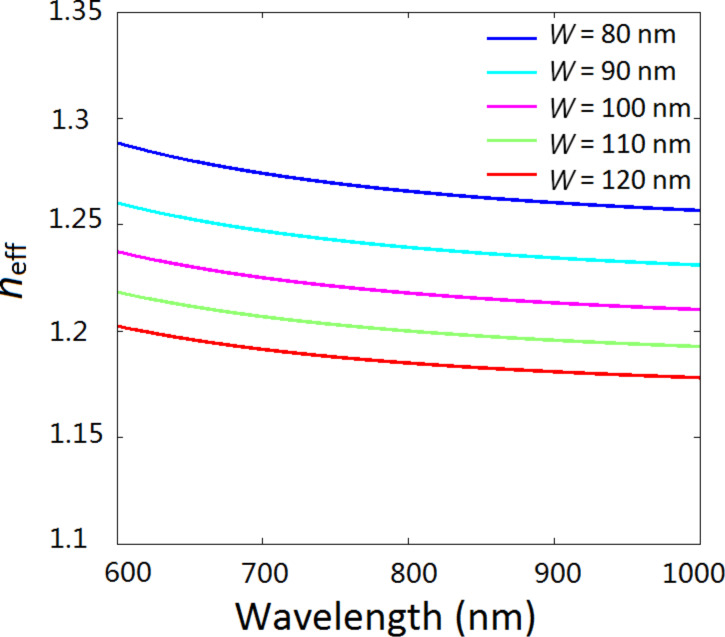
Effective refraction index *n*_eff_ as a function of the wavelength at different ring widths *W*.

Figure 4 presents the effective index *n*_eff_ as a function of the wavelength for different ring widths *W*. It is observed that the larger the width *W*, the lower the effective index *n*_eff_. Combining Equation 6 and Figure 4, we conclude that the resonance frequency of ring depends on the radii and the width of the ring. When the inner radius *r* increases from 35 to 75 nm, the effective length *L*_eff_ becomes larger, and at the same time the ring width decreases from 120 to 80 nm, which leads to bigger index *n*_eff_. As a result, both *L*_eff_ and *n*_eff_ increase with the increase of radius *r*. According to Equation 6, the corresponding resonance wavelength will become longer. The simulation shows that the Fano peak has a red-shift, which is consistent with the analysis.

Figure 3b shows the evolution of transmission as a function of the outer radius *R*. The inset shows the resonance wavelength λ_0_ as a function of *R*. With the increase of the outer radius *R* the transmission peak moves to longer wavelengths. Note that the red-shift in Figure 3b is smaller compared with that in Figure 3a. We attribute this phenomenon to different impacts of *L*_eff_ and *n*_eff_ on the transmission. When outer radius *R* increases from 135 to 175 nm, the ring width *W* increases from 80 to 120 nm. Bigger values of *W* will result in smaller *n*_eff_. On the contrary, the effective length *L*_eff_ increases with *R*. The blue-shift of the resonance wavelength caused by decreasing *n*_eff_ will partially offset the red-shift of the resonance wavelength caused by increasing *L*_eff_. This is the reason why the shift of the resonance peak in Figure 3b is smaller than that in Figure 3a.

**Figure 5:**
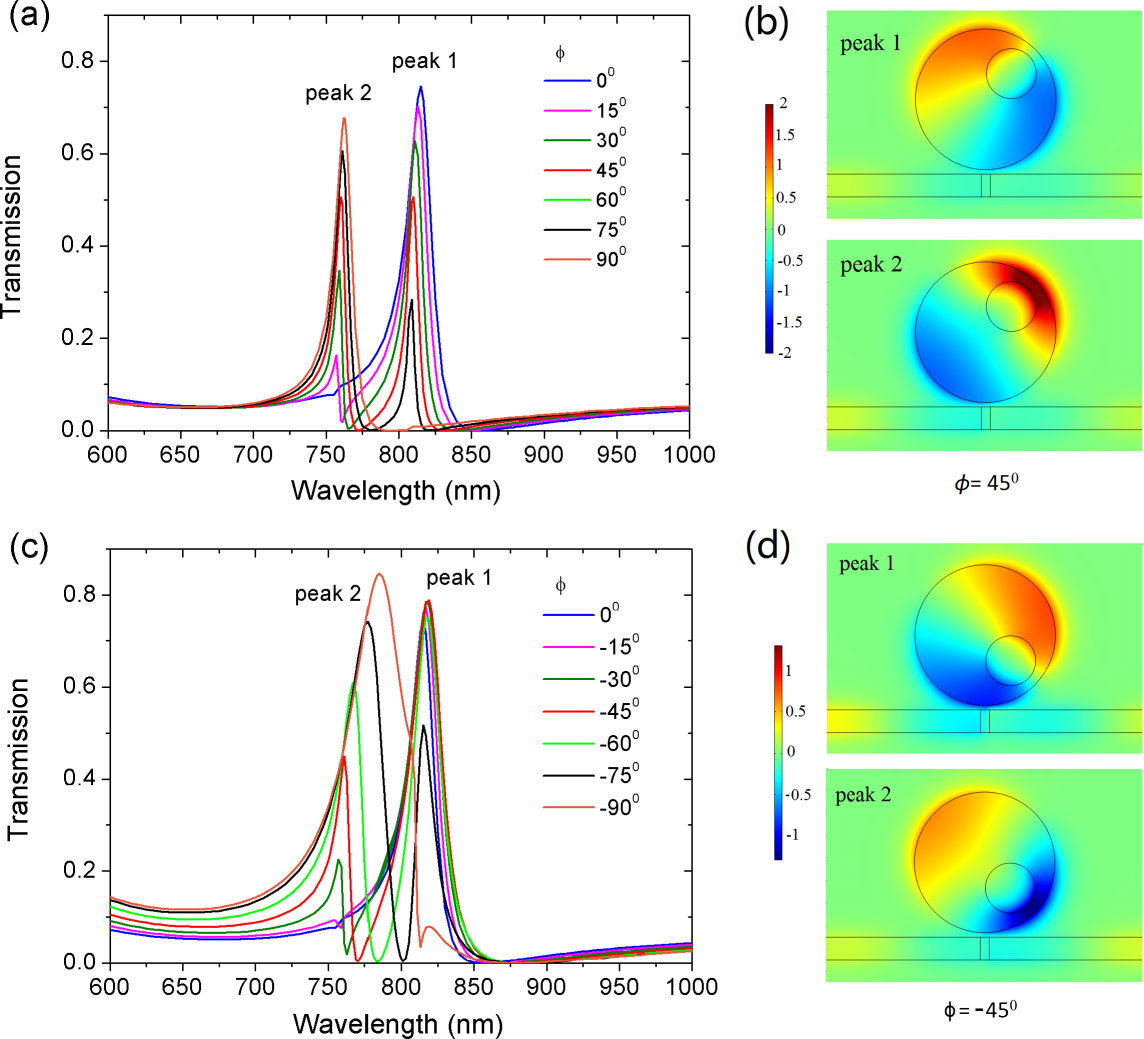
The evolution of transmission spectra with angle ϕ varying (a) from 0° to 90°, and (c) from 0° to −90°. The electric field distribution for peak I and II at (b) ϕ = 45° (810 nm and 760 nm), and (d) ϕ = −45° (819 nm and 761 nm). The other parameters are *R* = 155 nm, *r* = 55 nm, and the deviation distance *d* = 80 nm.

We have demonstrated that the ring is the key component for controlling the resonant frequency. In the following, we break the symmetry of the ring resonator and investigate the impact of symmetry-breaking on the Fano resonance. The symmetry-breaking is introduced by deviating center points *O* and *O*′. When the deviation distance *d* is greater than 0, a regular ring becomes an off-centered ring. Figure 5a displays the transmission spectra at *d* = 80 nm with varying angle ϕ. When ϕ is rotated counterclockwise, a new transmission peak 2 appears on the left side of the initial peak 1. With the increase of ϕ, the amplitude of peak 2 gradually increases while the amplitude of peak 1 gradually decreases. When ϕ reaches 90°, resonance 1 is totally suppressed and only resonance 2 exists. When ϕ is rotated clockwise from 0° to −90°, a similar phenomenon is observed (Figure 5c). A new resonance peak 2 appears and gradually increases its amplitude. Meanwhile, peak 1 is gradually decreasing. We note that the line widths of peak 2 in Figure 5a and Figure 5b differ. The electric field distributions for peak 1 and 2 at ϕ = 45° and −45° are shown in Figure 5b and Figure 5d, respectively. It is observed that peak 1 and peak 2 belong to the first mode order but their field patterns have different symmetry. It is the symmetry-breaking of the ring that leads to the change in the field distribution. The redistribution of field intensity would affect the coupling strength between ring resonator and waveguide. As a result, the linewidths of the resonance peaks differ.

**Figure 6:**
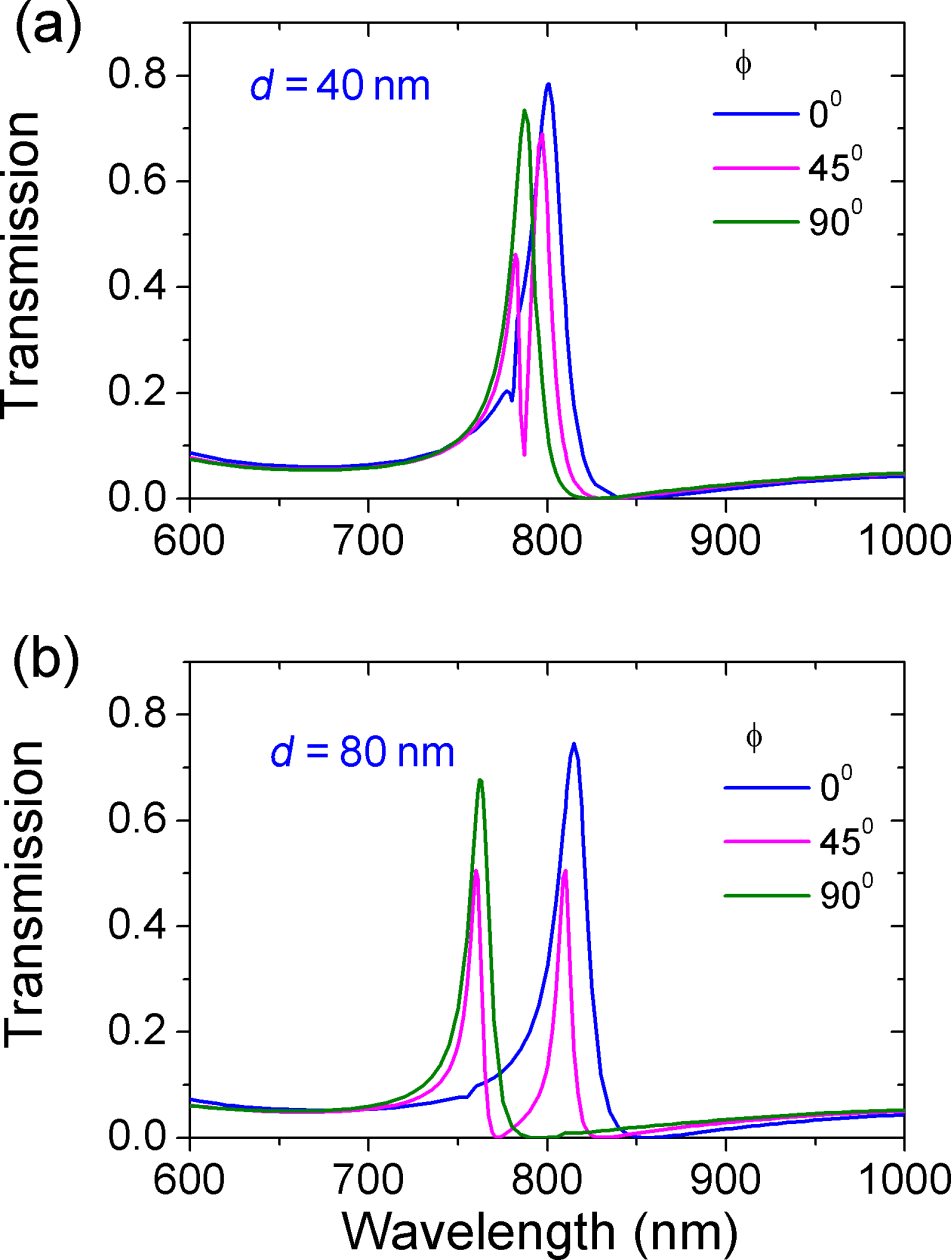
The evolution of transmission spectra with angle ϕ. The deviation distance is (a) *d* = 40 nm and (b) *d* = 80 nm. The other parameters are *R* = 155 nm and *r* = 55 nm.

Figure 6 shows the evolution of the transmission spectra at different deviation distances *d*. For clarity, only the angles ϕ are plotted. A new resonance peak 2 can be observed when *d* equals 40 and 80 nm. Comparing Figure 6a with Figure 6b, we find that as *d* increases, resonance 1 undergoes a red-shift while resonance 2 undergoes a blue-shift. The opposite shift leads to an increased wavelength interval between the two peaks at larger *d*. The reason is attributed to a greater asymmetry caused by a larger deviation distance *d*.

We have demonstrated in Figure 5 and Figure 6 that the dual resonances can be obtained by breaking the symmetry of ring resonator. Moreover, the resonance frequency and the number of peaks can be tailored through altering the degree of asymmetry, i.e., the deviation distance *d* and the deviation angle ϕ.

**Figure 7:**
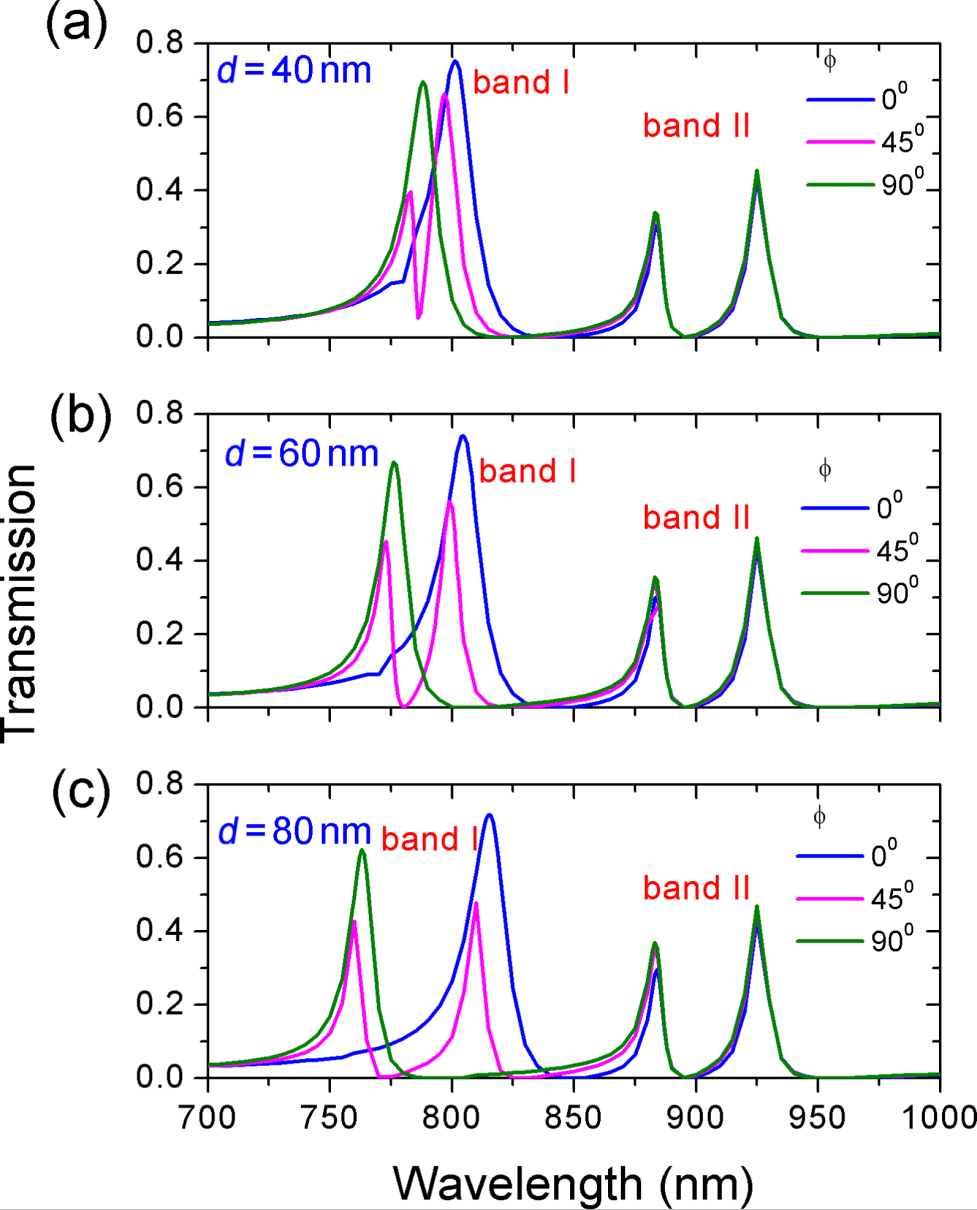
The evolution of transmission spectra as a function of the deviation angle ϕ of the first ring. The deviation distances are (a) *d* = 40 nm, (b) *d* = 60 nm, and (c) *d* = 80 nm. The other parameters of the initial ring are *R* = 155 nm and *r* = 55 nm. The deviation angle is ϕ = 45° and deviation distance is *d* = 80 nm. The parameters for of the second ring are *R*_2_ = 155 nm, *r*_2_ = 75 nm, *d*_2_ = 55 nm, and ϕ_2_ = −45°.

Now, another ring is placed at the other side of the waveguide with the gap distance *g* = 10 nm. The outer and the inner radius of the second ring are *R*_2_ = 155 nm and *r*_2_ = 75 nm, respectively. The deviation angle is ϕ_2_ = −45° and the deviation distance is *d*_2_ = 55 nm. Figure 7 shows the transmission spectra when the deviation angle ϕ of the first ring changes. Different deviation distances *d* are considered here. Two new peaks located at 880 and 925 nm are observed in addition to the original resonance peaks around 800 nm. For clarity, we refer to the new emergent resonance peaks as band II and to the initial resonance peaks as band I. In the presence of two resonators, the resonances of band I in Figure 7 show the same characteristic as the resonances in Figure 6. At ϕ = 0° and ϕ = 90°, only one peak is excited. At ϕ = 45° both peak 1 and peak 2 are excited. Furthermore, the larger the distance *d*, the more distinct is the wavelength interval between peak 1 and 2. However, the resonances of band II keep unchanged as ϕ varies. This different behavior demonstrates that band I stems from the coupling of the first ring and the waveguide. Therefore, band I depends on parameters of the first ring and is not influenced by the second ring.

**Figure 8:**
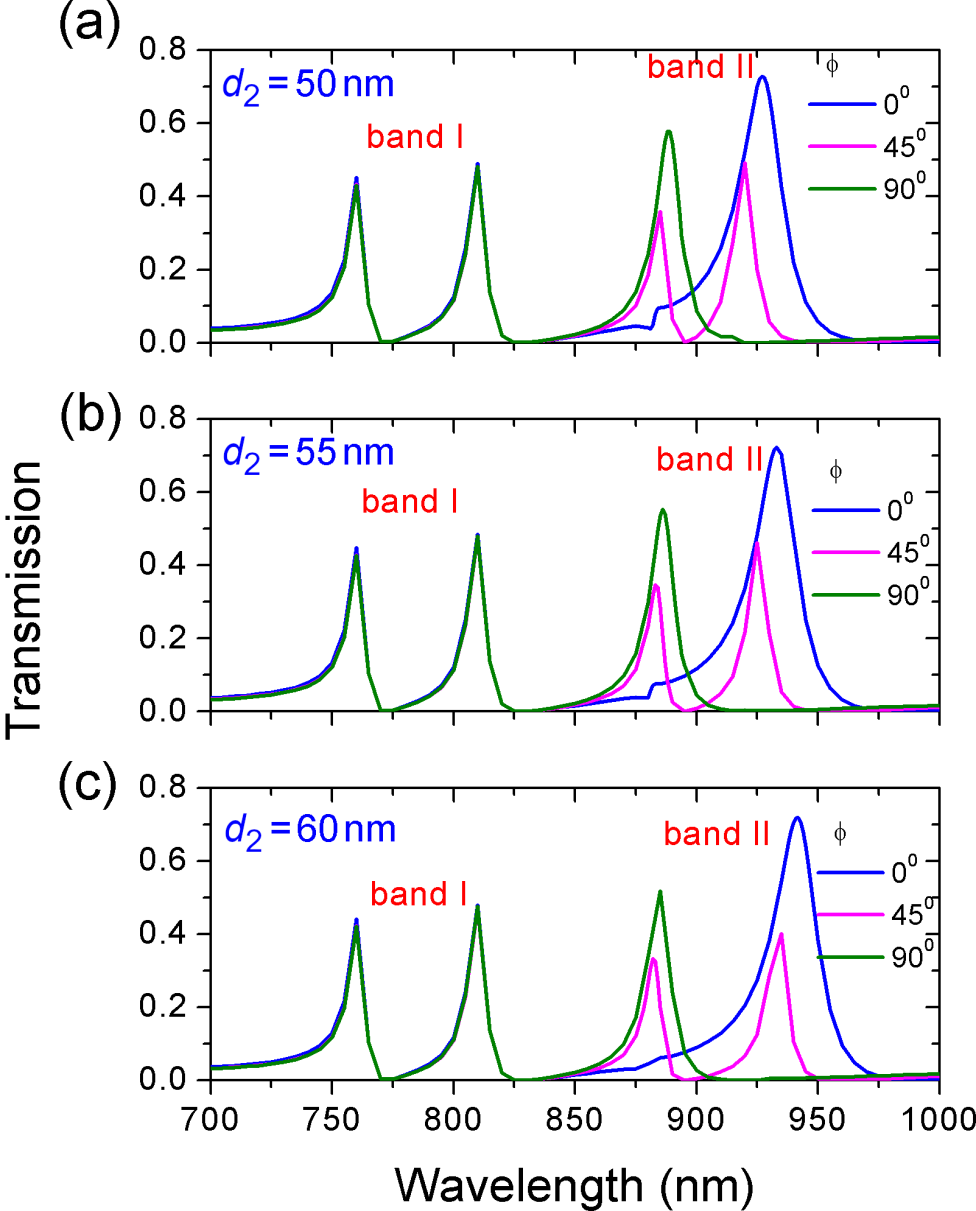
The evolution of transmission spectra as a function of the deviation angle ϕ_2_ of the second ring. The deviation distance *d*_2_ is (a) *d*_2_ = 50 nm, (b) *d*_2_ = 55 nm, (c) *d*_2_ = 60 nm. The other parameters of second ring are *R*_2_ = 155 nm, *r*_2_ = 75 nm. The parameters for initial ring are *R* = 155 nm, *r* = 55 nm, *d* = 80 nm, ϕ = 45°.

The impact of deviation angle ϕ_2_ of the second ring on the transmission was also investigated. The transmission spectra for varying ϕ_2_ at different deviation distances *d*_2_ are plotted in Figure 8. A similar phenomenon is observed. One or two resonances of band II appear as the deviation angle ϕ_2_ changes. Furthermore, the wavelength interval between resonance peaks 3 and 4 gets larger with increased *d*. However, the resonances of band I are independent of ϕ_2_. This behavior indicates that the resonances of band II originate from the coupling of the second ring and the waveguide.

**Figure 9:**
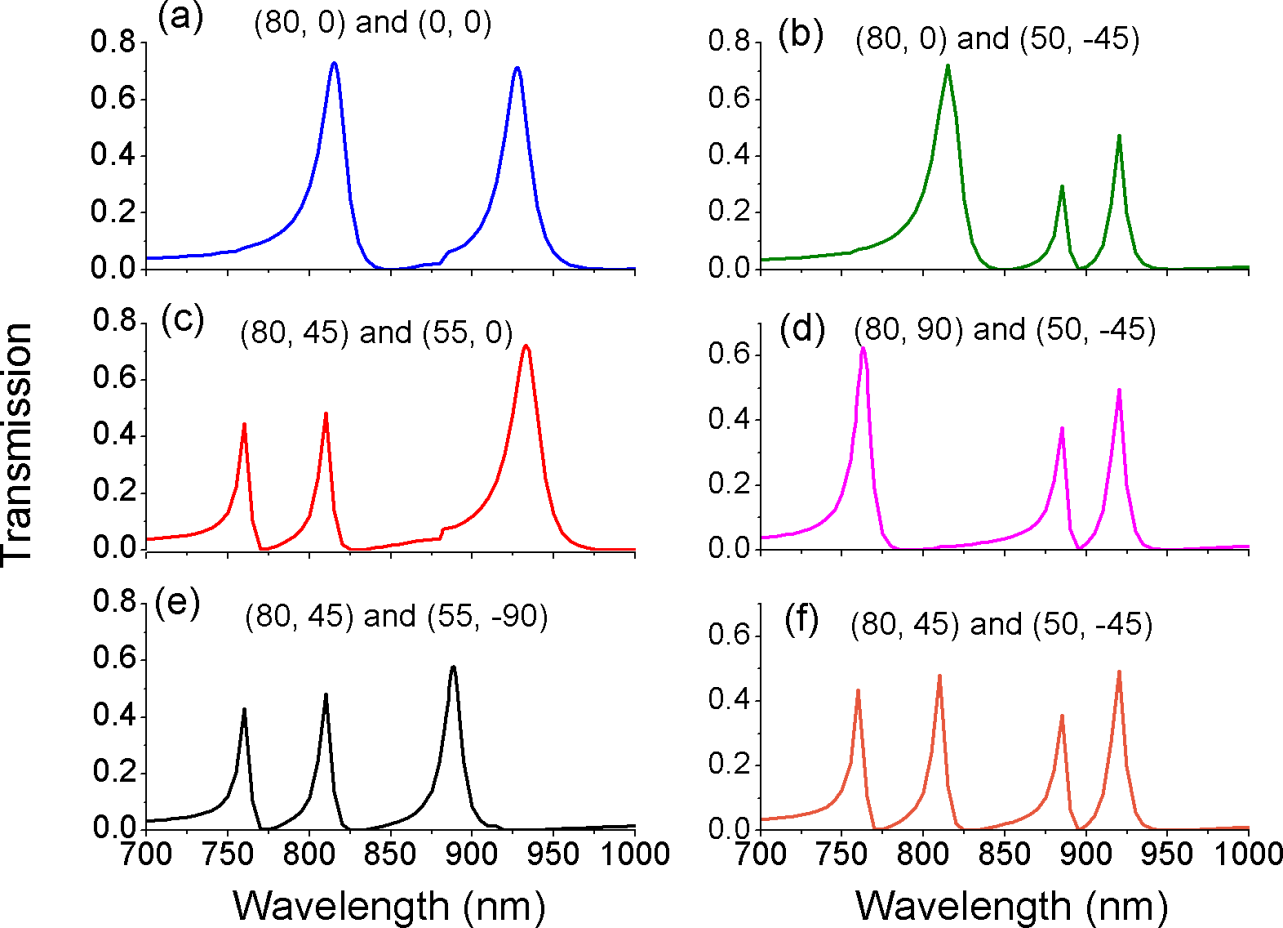
The evolution of transmission spectra with different parameters of two off-centered rings. (*d* [nm], ϕ [°]) and (*d*_2_ [nm], ϕ_2_ [°]) are (a) (80, 0) and (0, 0); (b) (80, 0) and (50, −45); (c) (80, 45) and (55, 0); (d) (80, 90) and (50, −45); (e) (80, 45) and (55, −90); (f) (80, 45) and (50, −45).

From Figure 7 and Figure 8, we know that the resonances of band I is determined by the first ring and the resonances of band II depend on the second ring. This different dependence provides a good opportunity to separately tune multiple Fano resonances. Figure 9 shows the transmission spectra with different parameters of the two ring resonators. The numbers in each subfigure stand for the value of (*d* [nm], ϕ [°]) and (*d*_2_ [nm], ϕ_2_ [°]). The outer and inner radii are constant. *R* = 155 nm, *r* = 55 nm, *R*_2_ = 155 nm, and *r*_2_ = 75 nm. It is seen that dual, triple, and even quadruple Fano-type transmissions are obtained by separately adjusting the deviation distance and deviation angle of each off-centered ring. In particular, there are triple peaks in Figure 9b–e but their resonant frequencies and frequency interval are not the same. This shows that multiple resonances can be arbitrarily tailored by changing the degree of asymmetry.

**Figure 10:**
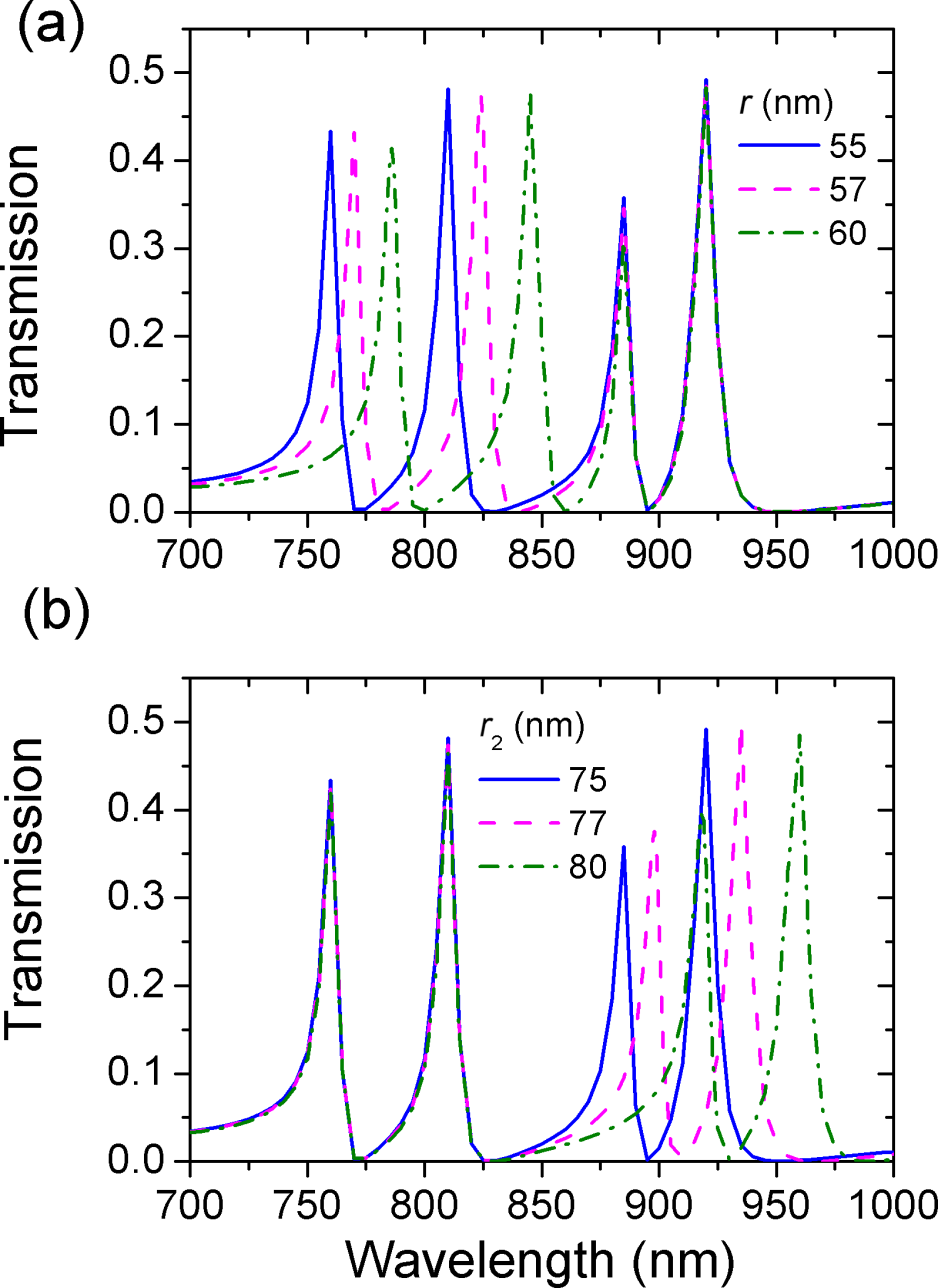
The evolution of transmission spectra with different radii. (a) Different values of *r* at *r*_2_ = 75 nm, (b) different values of *r*_2_ at *r* = 55 nm. The fixed parameters of the first ring are *R* = 155 nm, *d* = 80 nm, and ϕ = 45°. The fixed parameters of the second ring are *R*_2_ = 155 nm, *d*_2_ = 50 nm, and ϕ_2_ = −45°.

We have demonstrated that the radius of the rings is a key factor for Fano resonance in Figure 2 and Figure 3. The impact of the radius on multiple Fano resonances in the presence of two rings is further investigated. Figure 10 shows the evolution of transmission spectra as a function of the inner radii *r* and *r*_2_. Fano resonant peaks undergo a red-shift with the increase of the inner radii. Band I is most affected by *r*, and band II is most affected by *r*_2_. Multiple resonances can be independently tuned by changing the inner radii. Thus, one can regulate the frequency interval of resonance between band I and band II. Likewise, we find that the outer radius has an effect on the resonant frequency. In Figure 9 and Figure 10, we demonstrate that Fano resonances can be flexibly controlled by the structural parameters (*R*, *r*, *d*, ϕ) and (*R*_2_, *r*_2_, *d*_2_, ϕ_2_). These results are a great benefit to tunable integrated photonic devices.

## Conclusion

We have demonstrated multiple Fano resonances in a system in which off-centered ring resonators are coupled to a waveguide. By breaking the symmetry of the ring resonators, new resonant modes that are not obtained with regular concentric ring resonators are excited. Degrees of asymmetry are adjusted by changing the deviation distance and deviation angle of the asymmetric ring resonators. Dual, triple and even quadruple Fano-type transmissions are arbitrarily tailored. The frequency interval of the multiple Fano resonances can be tuned. The results may provide good guidance for designing flexibly tunable integrated photonic devices.
